# Contributions of the microbiome to intestinal inflammation in a gut-on-a-chip

**DOI:** 10.1186/s40580-022-00299-6

**Published:** 2022-02-08

**Authors:** Min Seo Jeon, Yoon Young Choi, Sung Jun Mo, Jang Ho Ha, Young Seo Lee, Hee Uk Lee, Soo Dong Park, Jae-Jung Shim, Jung-Lyoul Lee, Bong Geun Chung

**Affiliations:** 1 Department of Biomedical Engineering, Sogang University, Seoul, Korea; 2 Institute of Integrated Biotechnology, Sogang University, Seoul, Korea; 3 R&BD Center, hy Co., Ltd., Yongin-si, Korea; 4 Department of Mechanical Engineering, Sogang University, Seoul, Korea

**Keywords:** Gut-on-a-chip, Inflammation bowel disease, Microbiome, Shear stress

## Abstract

The intestinal microbiome affects a number of biological functions of the organism. Although the animal model is a powerful tool to study the relationship between the host and microbe, a physiologically relevant in vitro human intestinal system has still unmet needs. Thus, the establishment of an in vitro living cell-based system of the intestine that can mimic the mechanical, structural, absorptive, transport and pathophysiological properties of the human intestinal environment along with its commensal bacterial strains can promote pharmaceutical development and potentially replace animal testing. In this paper, we present a microfluidic-based gut model which allows co-culture of human and microbial cells to mimic the gastrointestinal structure. The gut microenvironment is recreated by flowing fluid at a low rate (21 μL/h) over the microchannels. Under these conditions, we demonstrated the capability of gut-on-a-chip to recapitulate in vivo relevance epithelial cell differentiation including highly polarized epithelium, mucus secretion, and tight membrane integrity. Additionally, we observed that the co-culture of damaged epithelial layer with the probiotics resulted in a substantial responded recovery of barrier function without bacterial overgrowth in a gut-on-a-chip. Therefore, this gut-on-a-chip could promote explorations interaction with host between microbe and provide the insights into questions of fundamental research linking the intestinal microbiome to human health and disease.

## Introduction

The intestine is a primary organ for digestion, absorption, and metabolism of the nutrients and drugs, establishing a protective barrier between the pathogen and other harmful microorganisms in a human body [1–3]. Furthermore, the intestine is the major site for cross-talk between the intestinal epithelium and commensal microbes of the gut microbiome [4, 5]. In general, the gut microbiome significantly contributes toward protection of the hosts against pathogenic incursions by enhancing the host defense mechanism [6]. For this reason, an imbalance between the composition and function of the intestinal microbes is associated with a number of diseases, such as inflammatory bowel disease (IBD), diabetes, obesity, cancer, and neurodegenerative diseases [7–9]. Thus, the human microbiome is appearing as a key player governing human health and the investigation of host-microbiome interaction emerges the substantial significance for drug development and disease treatment.

To study the interaction between gut microbiomes and host cells, there have been great efforts to develop experimental in vitro and in vivo models of the intestinal system that can be used to analyze intestinal physiology both in the present and absence of living gut microbiomes [10]. Over the past decades, the mimicking of the intestinal microenvironment has extensively been studied. The mammalian animal models [11, 12] and static transwell models [13, 14] are most commonly utilized to mimic the human intestinal system for investigations of human intestinal physiology and the microbiome. However, the animal model experiment is expensive, concerned about ethical and legal frameworks, and not fully scalable to human physiological responses. The experimental evidence showed that the static transwell models did not recapitulate the relevant aspects of the complex cellular diversity and dynamics (e.g., morphophysiology) of the human intestine, resulting in poorly reflection of in vivo physiology [15, 16]. Furthermore, the static transwell culture requires almost 3 weeks for the intestinal epithelial cells to differentiate into the intestinal lineage cells and fails to reveal some key intestinal differentiated functions [17, 18]. Recently, a three-dimensional (3D) human tissue surrogate, such as intestinal organoid (known as enteroid), has been emerged as a promising alternative due to its limitation of physiological relevance between living human intestinal system with intestinal cell lines [19–21]. However, one of the major drawbacks of the enteroids is the absence of mechanically active microenvironment of the living intestine (e.g., peristaltic motions or intralumenal fluidic flow) [22] that is critical for normal organ physiology as well as for development of Crohn’s disease and other intestinal disorders. Additionally, most of these in vitro intestinal models have not been adaptable to grow living microbes on the luminal surface of cultured intestinal epithelium due to overgrowth of microbes [23, 24], which usually dominate the co-culture system and induce human cell apoptosis within a day. This is a key problem, because the microbial symbionts normally contribute to intestinal barrier function, metabolism, and absorption of drugs and chemicals.

Recently, the development of the microfluidic technology has suggested an effective way to emulate the human intestinal environment, typically called “gut-on-a-chip”. This microfluidic platform includes controllable multiple system parameters (e.g., fluidic flow and oxygen concentration, mechanical deformation) to recapitulate the intestinal microenvironment situation. A number of researchers have developed gut-on-chip systems that could emulate intestinal environment to improve the cellular differentiation of human intestinal epithelial cells (Caco-2) and investigate the between the microbiome and host cells [25–27]. In most cases, the viability of the intestinal epithelium was better than transwell insert systems, thus allowing the long-term co-culture of microbiomes with host cells. However, one of the major limitations is that it relies on a complex device mechanically stretch intestinal epithelial cells rather than periodically wriggle like in vivo intestinal system. Moreover, the most of these studies were performed in the absence of other supporting cells and tissue types found within in vivo intestine system (e.g., blood vessel), which is important for disease modeling [28, 29]. Here, we developed the human gut-on-a-chip model that could emulate some relevant features and dynamic behaviors of the human intestinal tract that would overcome these limitations. First, we designed a three-channel microfluidic-based gut-on-a-chip to enable the co-culture Caco-2 cells with other supporting cells in the presence of physiologically relevant intestinal luminal flow, which could stimulate the differentiation of intestinal epithelial cell lineages of the small intestine, as previously described [30, 31]. Second, the osmotic pump was used to create flow in lumen fluids of the intestine and vessel, mimicking the stable fluidic flow of the intestinal lumen in vivo. We further analyzed the differentiation, microvilli, glycocalyx layer secretion, and barrier functions of the intestinal epithelial cells in a human gut on-chip. We also established a disease model of lipopolysaccharide (LPS)-induced intestinal injury and inflammation and assessed therapeutic effects of probiotics.

## Materials and methods

### Fabrication of the gut-on-a-chip with microelectrode arrays

The microfluidic-based gut-on-a-chip with microelectrode arrays was designed using Autocad (Autodesk, California, USA). The gut-on-a-chip master mold was made with SU-8 photoresist (MicroChem Corp., Massachusetts, USA) on a silicon wafer. SU-8 100 photoresist was spin-coated with 2000 rpm for 30 s on a 4-inch silicon wafer and baked at 65 ℃ for 20 min, 95 ℃ for 1 h, respectively. After soft baking, an UV light was exposed for 40 s with UV aligner (MDA-400LJ, Midas System Co. Ltd, Daejeon, Korea) and developed unexposed photoresist for 12 min. Microelectrode arrays were fabricated by E-beam physical vapor deposition. A positive photoresist was deposited on transparent soda-lime glass wafer (iNexus Inc., Gyeonggi, Korea) and developed by exposure to UV light using a mask aligner (Suss MA6 Mask Aligner, SUSS MicroTec AG, Garching, Germany). The chromium was sprayed onto the glass wafer to make a 5 nm thickness using an E-beam evaporator (ULVAC Inc., Kanagawa, Japan) and gold was then sprayed to have a 50 nm height. After sputtering, the unexposed portion of the photoresist was removed to form a metal electrode pattern. The polydimethylsiloxane (PDMS)-based gut-on-a-chip mold was prepared using a 10:1 mixture of a silicone elastomer and curing agent (Sylgard 184, Dow Corning Corp., Michigan, USA). The air bubbles were removed by vacuum for 30 min and PDMS-based gut-on-a-chip was polymerized at 80 °C for 1 h. The gut-on-a-chip mold were treated with in a plasma cleaner (Femto Science, Gyeonggi, Korea) to bond with microelectrode array substrates.

### Osmosis-driven fluidic flow set-up

PDMS cubic chambers with one cellulose membrane window were fabricated to make the osmotic pump using conventional protocols, as previously described [32]. The bonding of a cellulose membrane with a PDMS chamber was performed using the PDMS solution as adhesive glue. As a preliminary study, we conducted the osmosis experiments to evaluate the pumping capability of the osmotic pump. The deionized water was used as a buffer solution and polyethylene glycol (PEG) (Sigma-Aldrich, MO, USA; 2000 molecular weight) solution was used as a driving agent.

### Computational fluid dynamics (CFD) simulation

The microchannel modeling was performed with Autodesk Inventor (Autodesk Inc. USA) 3D CAD software and imported to COMSOL Multiphysics 5.5 (Comsol Inc., USA) software to conduct fluidic flow modeling. The fluidic fluid was assumed to be water and the microchannel wall settings were set to no slip condition. The extracellular matrix (ECM) channel was modeled using the physical properties of Collagen type I (Gibco™, Thermo Fisher Scientific, USA) with a density of 0.8536 kg/m^3^ and a viscosity of 6.6104 Pa∙s. The hexagonal-shaped micropillars with a width of 500 μm and a thickness of 150 μm were placed in a straight line between the microchannels. The height and width of the epithelial cell channel were defined in the range of 50 μm to 250 μm and 600 μm to 1000 μm, respectively, to determine the effect of channel dimensions on the fluid flow velocity profile and the wall shear stress acting between two channels. We calculated the fluid flow velocity profile by assuming the steady-state, single-phased laminar flow (SPF) with the inflow rates set to 0.5, 2.0, 3.5, 5.0, and 6.5 μL/min, respectively. Furthermore, the wall shear stress acting on the cell surface was calculated with the equation ${\uptau }_{\mathrm{w}}=\mu \bullet \dot{\gamma }$, where $\mu$ is the dynamic viscosity and $\dot{\gamma }$ is the shear rate (at the boundary of the cell surface). In our gut-on-a-chip system, the fluid flow through the microchannels was induced by osmotic pump. The flow rate is determined by the molar concentration of the PEG (Sigma-Aldrich, USA) solution. The average transferred volume was approximately 3.5 μL/min for a 0.18 M PEG solution, 7.0 μL/min for a 0.36 M PEG solution, and 10.5 μL/min for a 0.72 M PEG solution, respectively. To investigate the fluid velocity distribution inside the microchannel, we calculated the velocity in A-D sections of the central microchannel (Fig. 2D). The simulations were performed in the same method of the preceding conditions with respect to the molecular concentrations.

### Preparation and cell seeding on a gut-on-a-chip

The gut-on-a-chip was sterilized by autoclaving (120 °C for 30 min) and were dried in an oven. The gut-on-a-chips were coated with 1 mg/mL Poly-D-lysine (Sigma Aldrich, MO, USA) for overnight to improve cell adhesion and prevent the detachment of collagen type I gels. After coating, the gut-on-a-chip was rinsed with a deionized water more than three times, then placed in a dish at 80 °C in an oven at least 24 h. The collagen type I gel (Invitrogen, USA) with 2 mg/mL density was gently and slowly filled into the central channel. We placed the collagen gel-filled gut-on-a-chip in a CO_2_ incubator for 30 min to allow the collagen to gel. When the collagen solution was completely to gel, secondary coating was performed to epithelial cell channel with collagen and matrigel mixture for 15 min before seeding the Caco-2 cells. The human Caco-2 cells (ATCC clone HTB-37) were cultured in a modified Eagle’s medium with 10% fetal bovine serum, nonessential amino acids, L-glutamin, and penicillin–streptomycin in the absence of Calcium. The human umbilical vein endothelial cells (HUVECs) were cultured in EBM-2 basal medium with supplements and growth factors. Caco-2 cell and HUVEC suspension (100 μL, 1 × 10^4^ cells/ mL) were loaded into the microchannel using a micropipette. The cells in the suspension medium flowed into the microchannel by a gravity and were spontaneously trapped in the microchannel. As the cell suspension with homogenous density was applied in microchannel, the regular amounts of cells were allocated in each microchannel. We left the cells in the incubator for overnight without any treatment for stabilization of cells within the microchannel. After the cells were attached to the microchannel, the non-adherent cells were washed out. The outlet was connected with the flexible polyurethane tube.

### Immunofluorescence staining

For immunofluorescence microscopic analysis, the cells grown in the gut-on-a-chip were fixed with 4% (wt/vol) paraformaldehyde for 15 min, washed twice for 5 min with 0.1% bovine serum albumin (BSA) in phosphate buffer saline (PBS), and then permeabilized with 0.2% (vol/vol) Triton X-100 (Sigma Aldrich, MO, USA) for 20 min. After washing with 0.1% BSA in PBS, the cells were incubated with 3% (wt/vol) BSA blocking solution for 1 h. Subsequently, the cells were incubated with primary antibodies overnight at 4 °C, washed three times, incubated with secondary antibodies for 90 min, and washed three times with 0.1% BSA in PBS. The following antibodies were used for immunohistochemistry: mouse anti-ZO-1 (Invitrogen, USA, 1:200), rabbit anti-PECAM (Abcam, 1:500), mouse anti-MUC2 (Invitrogen, USA, 1:500), Alexa Fluor 594-conjugated phalloidin (Invitrogen, USA, 1:250), Alexa Fluor 488-conjugated Wheat Germ Agglutinin (Invitrogen, USA, 5 μg/mL, 1:200), Goat anti-mouse Alexa Fluor 488 (Invitrogen, USA, 1:1000), and Donkey anti-rabbit Alexa Fluor 594 (Invitrogen, USA, 1:1000). Samples were then incubated with 4′,6-diamidino-2-phenylindole dihydrochloride (DAPI; Molecular Probe, OR, USA) to visualize cell nuclei before taking confocal microscopic images (Olympus, Japan).

### Bacterial cell culture and live staining

*Lactiplantibacillus plantarum* HY7715 (HY7715) probiotic and *Bifidobacterium animalis spp. lactis* HY8002 (HY8002) probiotic were supplied by hy Co., Ltd. (Yongin-si, Korea). *Lactiplantibacillus plantarum* type strain ATCC14917 (ATCC14917) from the American Type Culture Collection (ATCC; Manassas, VA, USA) was used a reference strain to evaluate intestinal stability. *Lpb. plantarum* strains were grown in Man, Rogosa and Sharp (MRS) broth (BD, Franklin Lakes, NJ, USA) and *B. animalis spp. lactis* strain was grown in BL broth (MBcell, Seoul, Korea) at 37 °C for 24 h. Subsequently, the bacterial cells were harvested by centrifugation (4,000 rpm, 10 min, 4 °C), washed three times with PBS, and resuspended in cell culture media at 10^9^ CFU/mL before each assay. When bacterial cells were harvested by centrifugation (5000 g, 15 min) the cells were resuspended with 2 mL of raw EMEM. After suspension, the dye mixture of equal volumes (3 µL to each milliliter) of SYTO® 9 and propidium iodide (LIVE/DEAD® BacLight™ Bacterial Viability Kit, L7012, Thermo Fisher Scientific) was added. The cells with dye mixtures were incubated at room temperature in the dark for 15 min. After the two-time centrifugation and washing, they were inoculated in the Caco-2 cell microchannel.

### Inflammation study

To mimic the chronically inflamed microenvironment with engineered intestinal villi grown in a gut-on-a-chip was administrated the 15 μg/mL of lipopolysaccharide (LPS) (Invitrogen, USA) to the epithelial channel for 24 h. The ready to use eBioscience™ LPS (from *Escherichia coli* 026: B6) solution was directly diluted in raw EMEM to give an appropriate final concentration. Briefly, 5 days co-cultured intestinal models were treated 15 μg/mL of LPS concentrations for 24 h. After the inflammation induction, the pre-cultivated HY7715 probiotic was resuspended in a culture medium and cultivated in LPS-induced barrier dysfunction model at 37 ℃ for 3 days under the fluidic culture condition. After cell density was adjusted to 1 × 10^–8^ CFU/mL, HY7715 probiotic was introduced into the epithelium channel of the co-cultured model.

### Epithelial barrier analysis

The impedance was recoded using an alternating current (AC) voltage signal with an amplitude of 10 mV at a frequency range of 10 ~ 100 kHz (CHI 660E, electrochemical workstation). The transepithelial electrical resistance (TEER) was obtained by determining a suitable readout frequency from the impedance. TEER values are typically reported in units of Ω·cm^2^ and calculated as: TEER = R (Ω) × M_area_ (cm^2^), as previously described [33, 34] where, R is the cell-specific resistance across the cell layer, M_area_ is the effective area (0.0028 cm^2^). The magnitude of the impedances for these two electrode pairs were averaged for each session as average of |Z|. To obtain solely the information attributed to the growing cell layer, the magnitude of the impedances for each electrode pair of the measurements prior to cell seeding was subtracted from all the subsequent measurements during cell culture, resulting in the relative magnitude of the impedance [35].

### Statistical analysis

All results and error bars are represented as mean ± SEM. Statistical significance of differences between mean values was assessed with t-tests for unpaired data (GraphPad Prism software version 8.00 for Windows; GraphPad Software, Inc., San Diego, CA). A two-tailed unpaired student’s t-test was used to test significance between individual data sets as indicated. *p-value* less than 0.05 was considered statistically significant. All experiments were repeated at least three times to ensure reproducibility.

## Result and discussion

### Fabrication of a human gut on-chip model and CFD simulation analysis

**Fig. 1:**
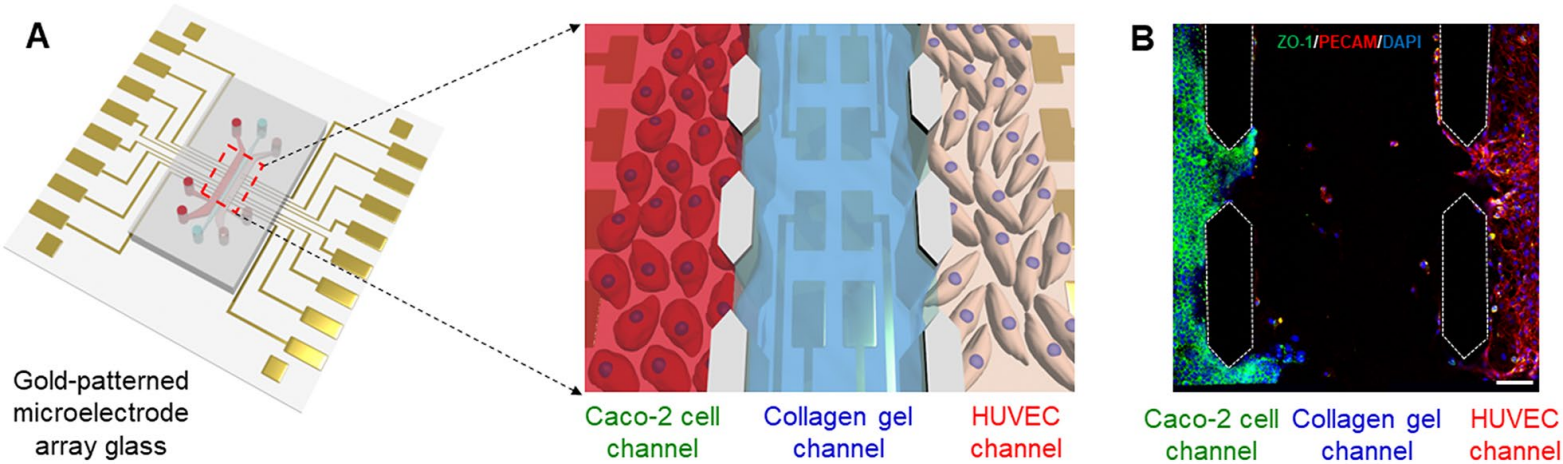
The human gut-on-a-chip with microelectrode arrays. **A** Schematic of the gut-on-a-chip. The left channel was used to establish an intestinal lumen using human epithelial Caco-2 cells. The right channel was employed to make a vascular lumen using HUVECs. The continuous flow of the culture medium was introduced by an osmotic pump. **B** Representative immunofluorescence results showing that ZO-1-positive Caco-2 cells and PECAM-1-positive HUVECs were located in the left and right channel of a gut-on-a-chip separated by collagen type I gels. Scale bar is 100 μm

**Fig. 2:**
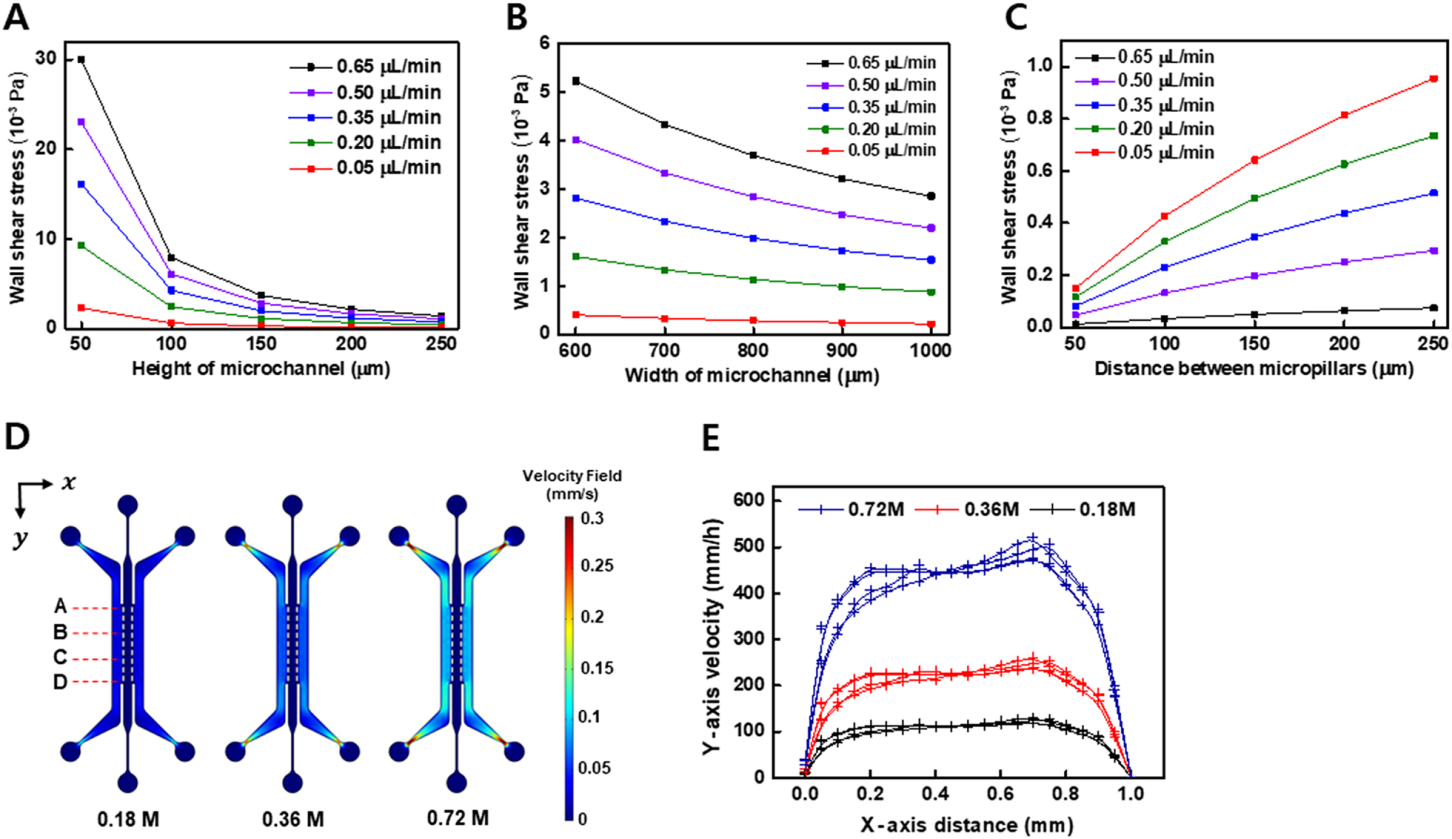
Simulation of a gut-on-a-chip under various fluidic flows and geometric conditions. **A** Wall shear stress is calculated with respect to the height of the microchannel. **B** Wall shear stress is calculated with respect to the width of the microchannel. **C** Wall shear stress is calculated with respect to the distance between the micropillars. **D** Flow speed distribution in the cell culture microchannel. CFD simulations were performed to analyze the velocity at various concentrations of 0.18 M, 0.36 M, 0.72 M PEG solutions. **E** Velocity distribution inside the microchannel is calculated in a section of **A**–**D**

To investigate the interactions between microbiomes and human Caco-2 cells in a human intestinal microenvironment, we developed a microfluidic-based gut-on-a-chip embedding with microelectrode arrays (Fig. 1A). The gut-on-a-chip consisted of three parallel microchannels (500 μm wide, 10 mm long, 150 μm high) separated by the hexagonal-shaped micropillars: two stromal cell culture channels which could mimic the epithelium or endothelium layer and one central channel filled with collagen type I gel (Fig. 1A, right). We used the three parallel microchannels separated by collagen gel channel without any polymeric membrane, showing that it was relatively easy to fabricate. By using an osmotic pump, the culture medium was perfused at simulated flow rates through each microchannel, mimicking the fluidic flow and associated shear stress on the cell surface in the human intestinal lumen and the blood vessels in vivo. We co-cultured Caco-2 cells and HUVECs separated by collagen gels in a gut-on-a-chip (Fig. 1B). Prior to the experiment, the flow dynamics of the gut-on-a chip were performed in CFD simulation to assess the correlation between wall shear stress and the dimensions of the microfluidic channel (Fig. 2). Within the microchannel, the flow velocity $u$ is dominated by the homogeneous, incompressible Naiver-Stokes equation and continuity equation:

$$\rho \left(u\bullet \nabla \right)u=\nabla \bullet \left[-p{\rm I}+\tau \right]+F$$

$$\rho \nabla \bullet u=0$$

where $\rho$ is the fluid density, $u$ is the velocity field, $\nabla$ is the divergence, $p$ is the pressure, $\tau$ is the Stoke’s stress ($\tau =\mu (\nabla u+\nabla {u}^{T})$), $\mu$ is the dynamic viscosity, and $F$ is the volume force. In consequence, the wall shear stress acting on the epithelial cell channel was derived from the previously obtained velocity field. Assuming an isotropic Newtonian flow in the channel, the shear stress inside the shear stress inside the microfluidics can be calculated by the following equation:

$$\tau \left(\overrightarrow{u}\right)=\mu \nabla \overrightarrow{u}$$

where μ is the dynamic viscosity, and $\nabla \overrightarrow{u}$ is the gradient of velocity field which is also called as wall shear rate. The average value of the wall shear stress was acquired from the boundary of the epithelial cell channel in which the lactic acid bacteria were cultured. The height and width of epithelial cell channel and the distance between the micropillars are set as parameters. The dimensions of the parameters were set in the ranges of 50 to 250 μm, 600 to 1000 μm, and 50 to 250 μm, respectively. It is important to find the optimal microchannel dimension for the well growth of lactic acid bacteria. Since the wall shear stress acting at the boundary surface is a crucial factor for the cell growth [36], the correlation between channel dimension and wall shear stress needs to be considered to determine optimal channel dimension. Simulating with respect to the microchannel height, the wall shear stress rapidly decreased as the channel height was increased (Fig. 2A). The average wall shear stress values for the channel height of 0.05, 0.2, 0.35, 0.5, and 0.65 μm at a flow rate of 0.35 μL/min were 16.16, 4.28, 1.99, 1.17, and 0.76 10^–3^ Pa, respectively. The lower the height of microchannels, the higher the wall shear stress. From the results of CFD simulation for the microchannel width, the wall shear stress was proportional to the microchannel width (Fig. 2B). Within a fixed flow rate, the changes in microchannel height and width leaded to variation in channel volume, which could affect the flow velocity. Simulating with respect to micropillar distance, the wall shear stress was increased with the micropillar distance (Fig. 2C). The flow rate was simulated in the range between 0.05 and 0.65 μL/min for each parameter. All three simulations showed the same tendency for wall shear stress to increase in proportion to the flow rate, because the laminar flow shared the same streamline in the identical geometry [37]. The distribution of the flow velocity in the channel at the different concentrations of PEG solutions were calculated (Fig. 2D). In the case of flow driven by 0.18 M PEG solution, the velocity was relatively uniform over most of the channel, whereas the flow with 0.36 M and 0.72 M PEG solutions exhibited wide variations between the upper and lower flows. The velocity at a position of A, B, C, and D is plotted in Fig. 2E, which shows that the mean velocities driven by 0.18 M, 0.36 M, and 0.72 M PEG were 99.55 mm/h, 197.16 mm/h, and 394.21 mm/h, respectively. At 0.36 M PEG concentration, the flow speed was increased and the colonies of microbiome were detached from the differentiated Caco-2 cells. In contrast, the flow speed was decreased at a 0.09 M PEG concentration, showing that the colonies of microbiome were rapidly grown. On the basis of these results, we optimized a flow speed of 0.18 M PEG solution, which could produce the most stable fluidic culture model in subsequent experiments.

### Effect of fluidic flow and endothelial cell on differentiation of epithelial cells in gut on-chip

**Fig. 3:**
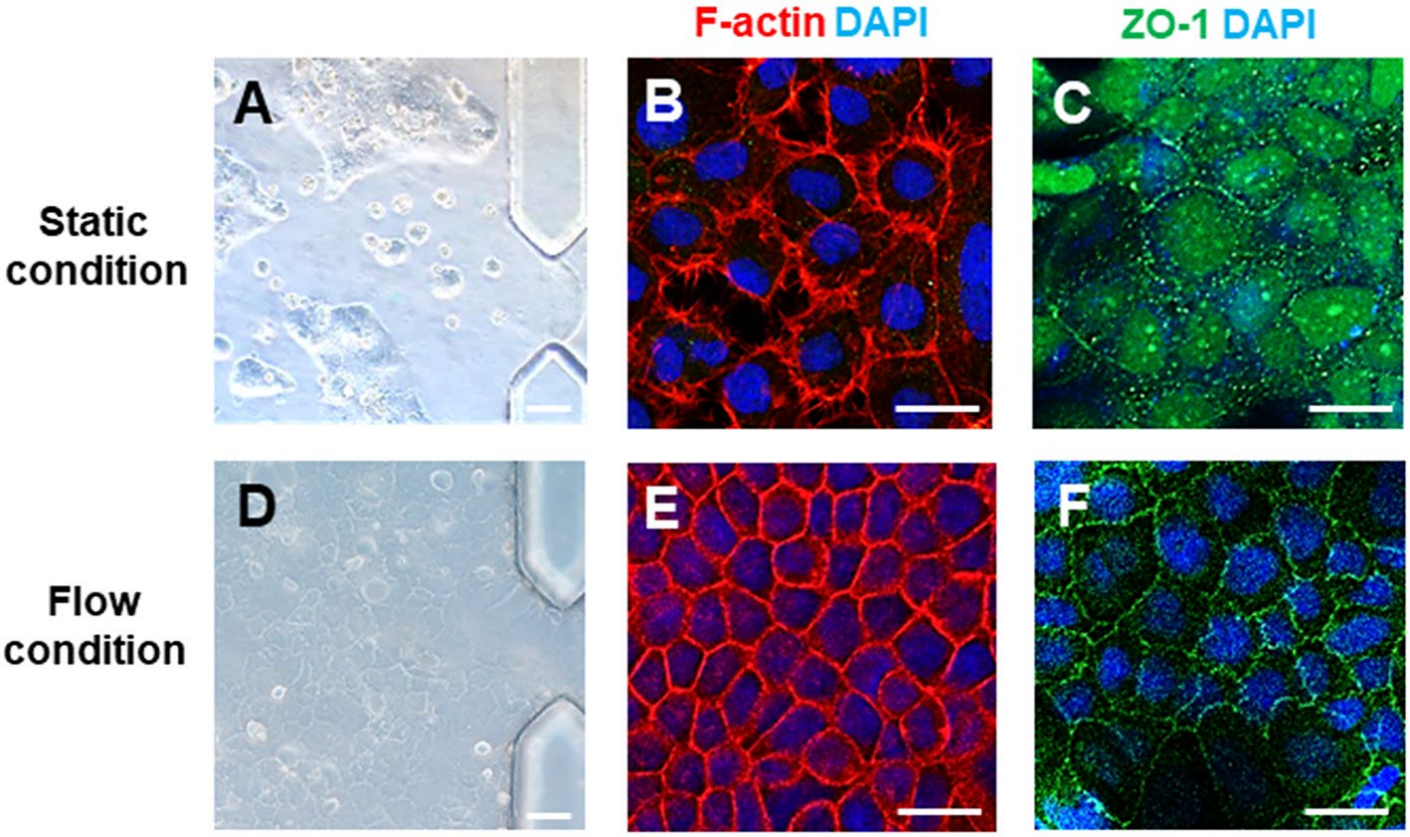
Morphology of the Caco-2 epithelial cells cultured in a static culture condition and in the gut-on-a-chip with microfluidic flow for 5 days. **A** and **D** are phase contrast views of Caco-2 cells monolayer. **B** and **E** show the distribution of the cytoskeleton protein, F-actin, in the Caco-2 cells monolayers. **C** and **F** show the distribution of the tight junction protein, ZO-1, in the Caco-2 cells monolayers. Scale bars are 25 μm

**Fig. 4:**
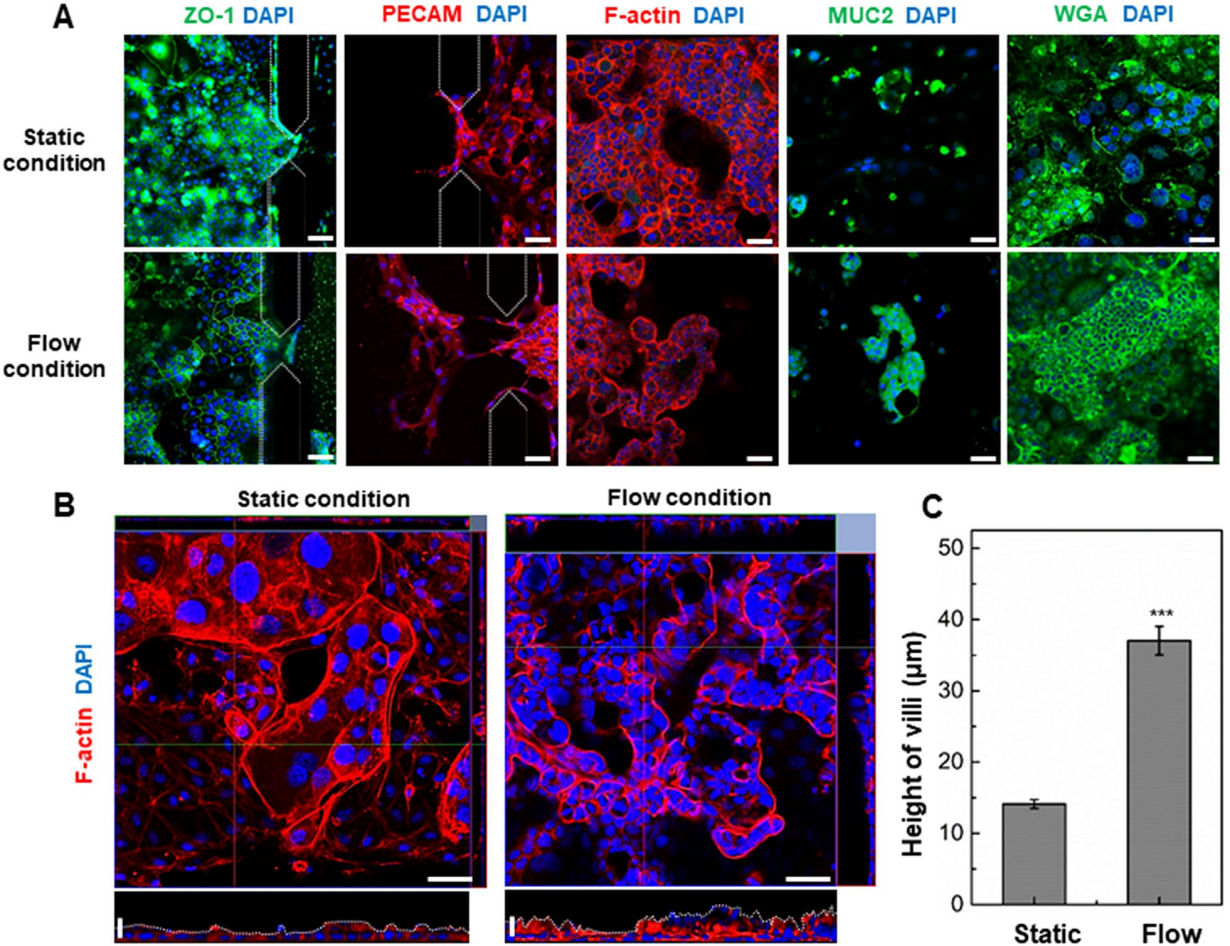
Morphology of the Caco-2 epithelial cells co-cultured with HUVECs in a static and flow culture condition for 5 days. **A** Morphological analysis of intestinal specific proteins under a static and flow condition. Scale bars are 50 μm. **B** Morphological analysis of polarized columnar epithelium. Fluorescence confocal micrographs (vertical cross-sectional views at 5 days after onset) show a vertical cross-section of the epithelium highlighting cell shape and polarity. Horizontal scale bars are 50 μm and vertical scale bars are 25 μm. **C** The average height of Caco-2 cells grown in the gut-on-a-chip without and with fluidic flow (***p < 0.001)

We first explored whether the shear stress generated by luminal fluidic flow above the epithelium is responsible for induction of the epithelial morphogenesis. To do this, Caco-2 cells were grown either in a static culture or microfluidic culture condition (Fig. 3). After 5 days of cultivation, the Caco-2 cells remained viable in the both fluidic and static conditioned gut-on-a-chip, which was higher cellular density than in the static conditions (Fig. 3A, D). We analyzed the fluidic flow effect on epithelial barrier integrity in Caco-2 cells confirmed by the immunostaining of junctional ZO-1 and the labeling of the actin cytoskeleton, F-actin. When cultured in a fluidic condition, the actin cytoskeleton showed a continuous ring appearance between adjacent cells, whereas the actin staining appeared the discontinuous and less ordered in a static condition (Fig. 3B, E). In addition, the immunostaining data showed the bright signals of the tight junction protein (ZO-1) at the edge of cells cultured in the fluidic culture condition, suggesting that the Caco-2 cells could form the confluent polygonal epithelial monolayers with well-developed tight junctions in a fluidic culture condition, much tighter than cells in a static culture condition. (Fig. 3C, F). Nevertheless, the cultured human intestinal epithelial cell alone spontaneously was not formed highly polarized epithelium and mucus secretion cells. To more effectively ameliorates the mimic in vivo intestinal system and differentiation of the Caco-2 cells, we co-cultured Caco-2 and HUVECs in a gut-on-a chip which separated with 2% collagen gels (Fig. 4). In the same flow conditions, one of the most noticeable changes in a co-culture model, the expression of polarized and differentiated columnar epithelium (Fig. 4A down panel, F-actin) appeared similar form to living in vivo intestinal villi, as previously described [38]. To investigate the effect of fluidic flow on the differentiation, the 3D projections in the Z-stack of the confocal images were used for quantifying the epithelial layer height (Fig. 4B). The epithelium layer differentiated by Caco-2 cells cultured in a gut-on-a-chip showed the 37.04 ± 2.38 μm height of the villi, while those under static conditions were only 14.11 ± 0.74 μm, respectively (Fig. 4C). Another important characteristic to consider when developing in vitro models of the gut is the presence of a mucus layer, which is most abundant structural protein of the gastrointestinal mucus layer [39]. We evaluated Mucin 2 (MUC2) visualized via immunofluorescence (Fig. 4A down panel, MUC2). We confirmed that the Caco-2 cells cultured in a fluidic culture condition generally produced more MUC2 expression as compared to cells grown in a static culture condition. They were located at the tips of the villi-like structures. Mucin are known to protect the underlying epithelium from mechanical stresses [40], which is expected to be highest at the tip of the villi-like structures under fluidic culture conditions. These results are consistent with previous studies combining Caco-2 and other gastrointestinal cell lines with fluidic flow that reported improved mucus production in response to mechanical stimulation [31, 39]. The Caco-2 cells acted as absorptive enterocytes [41, 42], and formed a continuous, planar epithelial monolayer in transwell inserts after 3 weeks of culture, as previously described [14]. However, they could not exhibit similar in vivo intestinal cell differentiation when grown under static culture conditions. In contrast, in our gut-on-a-chip system, the Caco-2 cell monolayer co-cultured with HUVECs spontaneously initiated villus morphogenesis within 5 days when cultured in the presence of fluidic flow and other supporting cells mimicking the physical microenvironment experienced by in vivo intestinal system. Glycocalyx, an efficient defense system for protecting the epithelium from pathogens [43], was evaluated by expression of WGA-Alexa488, which could bind to sugar residues on cellular surface. A marked increasing in WGA-Alexa488 binding was observed in a flow culture condition as compared to a static condition, suggesting that the flow culture condition could improve the epithelial cell differentiation. As expected, the expression of glycocalyx was influenced by a fluidic condition, indicating that flow condition promoted the differentiation of Caco-2 cells. Thus, our results suggest that the mechanical factors of the fluidic flow and cellular components are the crucial microenvironment cues that can drive more complete intestinal differentiation process than local stromal factors [44]. Furthermore, these results indicated that Caco-2 cells cultured in the presence of continuous flow required a shorter time to polarize and differentiate.

### Effect of fluid flow and endothelial cell on barrier integrity of epithelial cells in gut on-a-chip

**Fig. 5:**
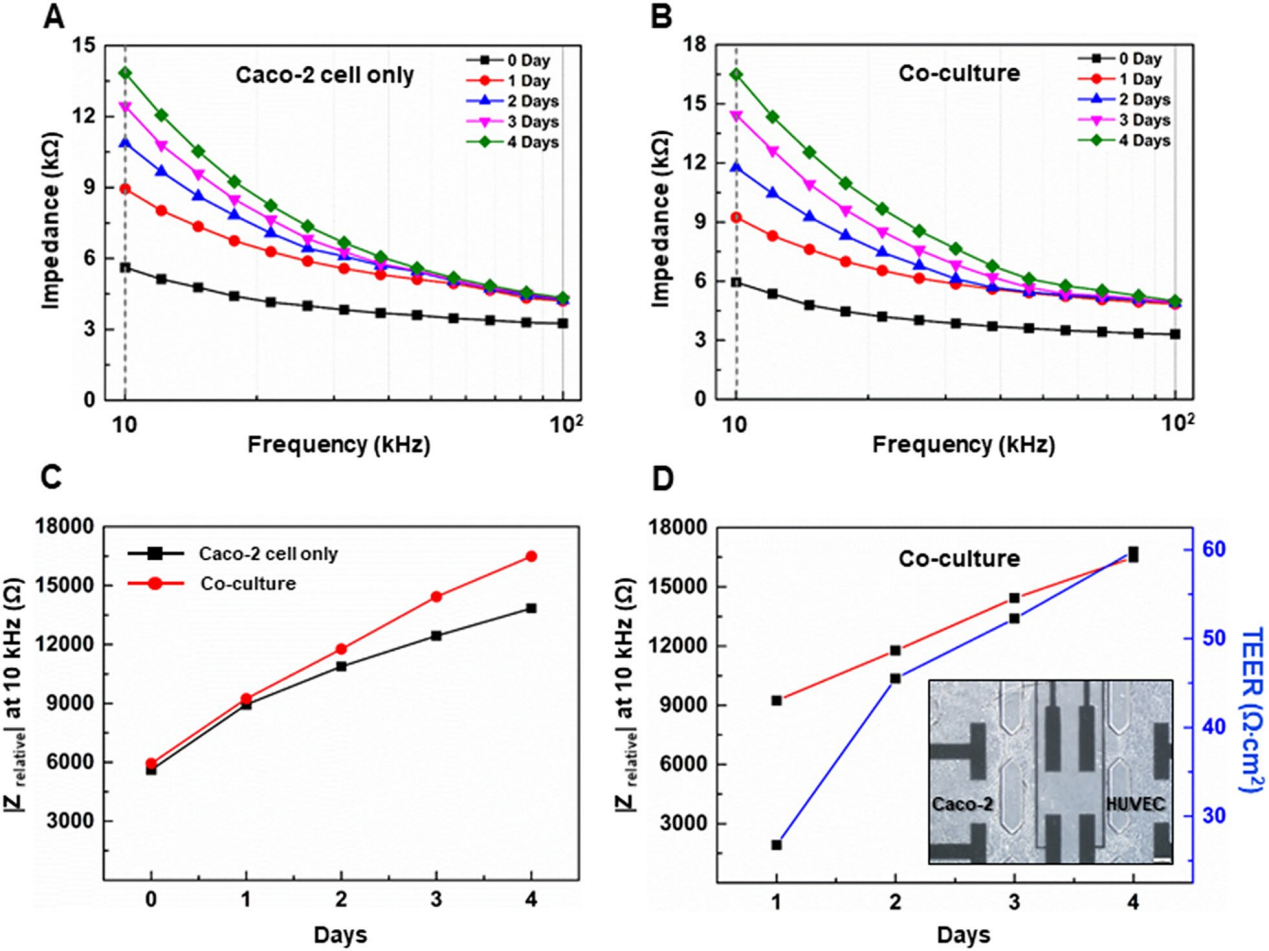
Impedance analysis in a human gut-on-a-chip. Impedance spectra showing the development of an intestinal epithelial barrier cultured with **A** Caco-2 cell only and **B** co-culture with Caco-2 cells and HUVECs. **C** Epithelial resistance with respect to culture days, showing that the resistance of the co-culture model was increased with culture days. **D** Epithelial impedance versus calculated TEER analysis in the co-cultured model

Caco-2 cell only cultured model and co-cultured model were exposed to fluidic flow for 4 days in a gut-on-a-chip and the differentiation process was evaluated using impedance spectrometry (Fig. 5). The impedance spectra of the Caco-2 cell only cultured model and co-cultured model in a gut-on-a-chip showed that the maximum difference in the impedance spectra was observed at 10 kHz (Fig. 5A, B). The measurement of day 0 before cell seeding was subtracted from all subsequent measurements (resulting in the |Z_relative_|) to confirm the change in impedance attributed to the cell layer. The relative impedance |Z_relative_| at 10 kHz was monitored in a Caco-2 cell only cultured model and co-cultured model on a gut-on-a-chip over 4 days (Fig. 5C). This non-invasive method can be applied to living cells and allows them to be monitored during growth and differentiation, since their morphological changes can be described by variations in impedance measurements [45]. In particular, it is important for monitoring the growth of cellular extrusions like microvilli [46]. In a gut-on-a-chip with Caco-2 cell only cultured model and co-cultured model, the measured impedance kept increasing during all 4 days, showing that the Caco-2 cell only cultured model and co-cultured model was 13 kΩ and 16 kΩ, respectively. The shear stress induced by the fluidic flow has the effect of the mechanotransduction on several endothelial molecular pathways through activation of membrane-bound receptors, leading to the production of the tight junction proteins (e.g., ZO-1). It modulated the cytoskeletal structure to promote the cell reorientation and restructuring [47, 48]. Hereby, the resistance of the sensor surface covered by the cells tends to be increased due to cell proliferation and spreading [49]. Since the current has to flow through the cells, the resistances between the measuring electrodes can keep increasing and the TEER barrier resistance can keep increasing [49]. The relative impedance |Z| and TEER were characterized by an increase in measured cell layer resistance for co-cultured model on a gut-on-a-chip (Fig. 5D). The calculated TEER value was reached up to 59 Ω∙cm^2^ over 4 days of culture. The previous study has reported a time-dependent barrier formation of human intestinal cells developing from 20 to 60 Ω∙cm^2^ in a static culture [50]. This value was comparable to our TEER-measurements. However, our co-cultured model on a gut-on-a-chip showed increase in the barrier strength within 5 days of fluidic flow culture. These results demonstrated that the application of fluidic shear stress and endothelial cells enhanced the cellular differentiation and barrier formation of the intestinal cells.

### Host cell and probiotic co-culture on gut-on-a-chip

**Fig. 6:**
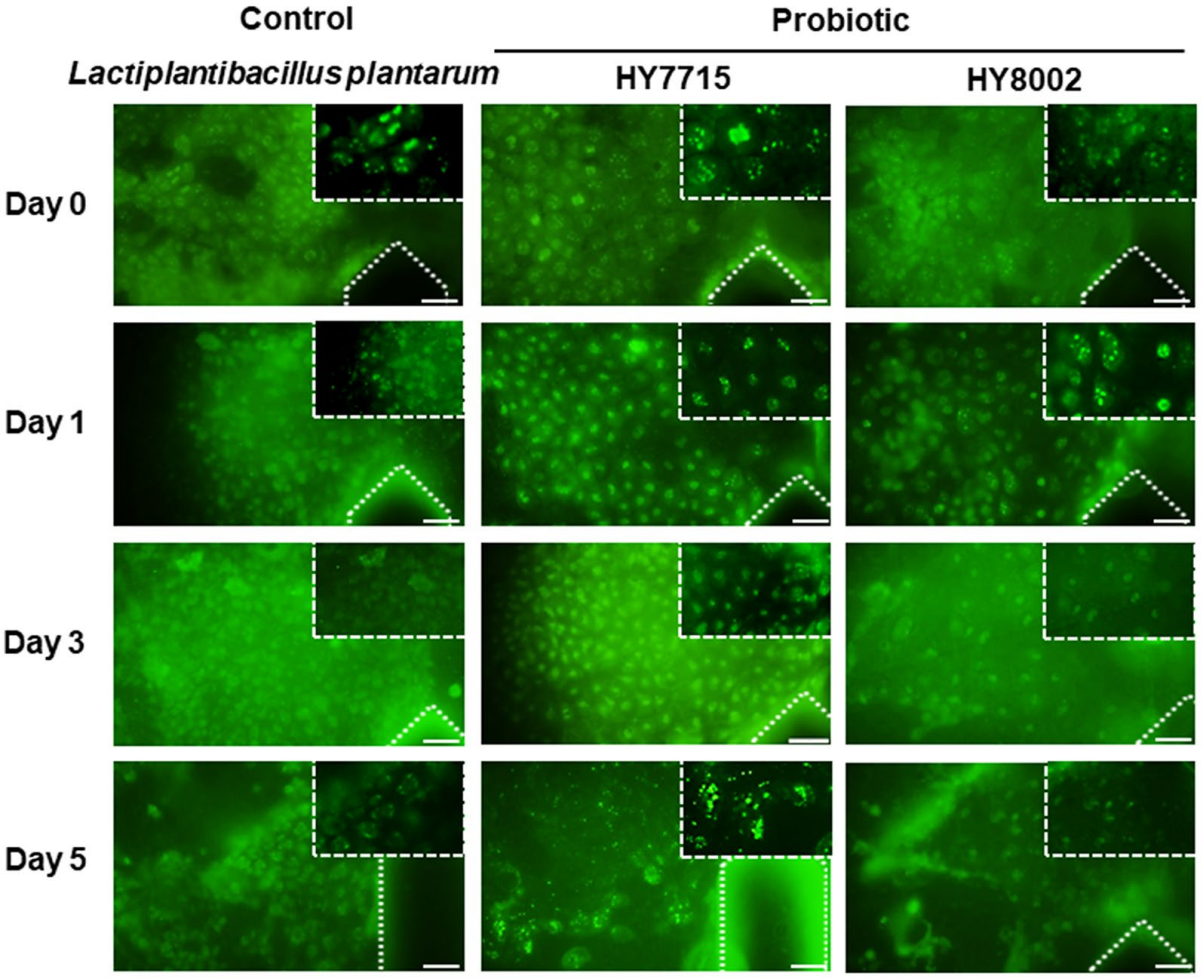
Microbial co-culture on a human intestinal epithelial monolayer in a gut-on-a-chip. Bacteria were cultured on the surface of a Caco-2 cell monolayer grown within a gut-on-a-chip (*Lactiplantibacillus plantarum* as a control, *Lactiplantibacillus plantarum HY7715* probiotic and *Bifidobacterium animalis spp. lactis HY8002 probiotic*; seeding density 1 × 10^8^ cells/mL). Fluorescence views from above of probiotics and Caco-2 cell co-cultured for 5 days and viewed at low and high (white-dotted rectangle) magnification, which shows microcolonies of the bacteria (green spots) that remain tightly adherent to the apical surface of the Caco-2 cell monolayer after exposure to continuous fluidic flow. Scale bars are 100 μm

The adhesion of commensal bacteria to host cells is considered as an appropriate parameter to determine the colonization potential of a probiotic strain [51]. However, as the bacterial overgrowth occurs rapidly compromising the epithelium, it is impossible to expose these cells to living microbiome in long-term culture [30]. Thus, the establishment of the stable symbiosis between the epithelium and resident gut microbiome as observed in the normal intestine is crucial to maintain the normal epithelial differentiation and restrain microbial overgrowth in the intestine in vivo [52]. In a present study, we leveraged our gut-on-a-chip to maintain the probiotics. In this study, a commercial probiotic, *Lactiplantibacillus plantarum* was used as a control, *Lactiplantibacillus plantarum* HY7715 probiotic and *Bifidobacterium animalis spp. lactis* HY8002 probiotic were employed. Blocking the fluidic flow for the first 2 h, bacterial cells were allowed to adhere on the apical surface of villi. After 2 h, the physiological relevant flow was resumed through the microchannels to remove un-colonized gut bacteria and supply nutrients to both bacterial and villus epithelial cells. When a non-pathogenic laboratory strain of green fluorescent stained probiotics was allowed to adhere to the apical (luminal) surface of villi for 2 h under static conditions, these bacteria cells were subsequently colonized and spontaneously inhabited regions (Fig. 6). When the bacteria were cultured on the villus epithelium layer under a flow condition (21 μL/hour), we observed the colonized stable form until day 1 in all probiotic groups. However, *Lpb. plantarum* and HY8002 probiotic seemed detached from the villi and washed out after 3 days when cultured under flow conditions as compared to HY7715 probiotic, although the luminal flow was maintained constant. All species showed adhesion to the used epithelial layer, however, the adhesion level of HY7715 probiotic was greater to epithelial layer even in day 5. These results were concurred with the finding by *Schillinger *et al*.,* who showed that the adherence of diverse probiotic strains varied among strains [53]. In addition, *Gopal *et al., has reported the higher affinity of *L. acidophilus* and *L. rhamnosus* strains to HT29-MTX cells than HT29 and Caco-2 cells [54]. Nevertheless, we need to optimize the incubation time and volumetric flow rate for attachment to the surface of villi.

### LPS-induced intestinal damage responses and evaluation of barrier protection effect of probiotics

**Fig. 7:**
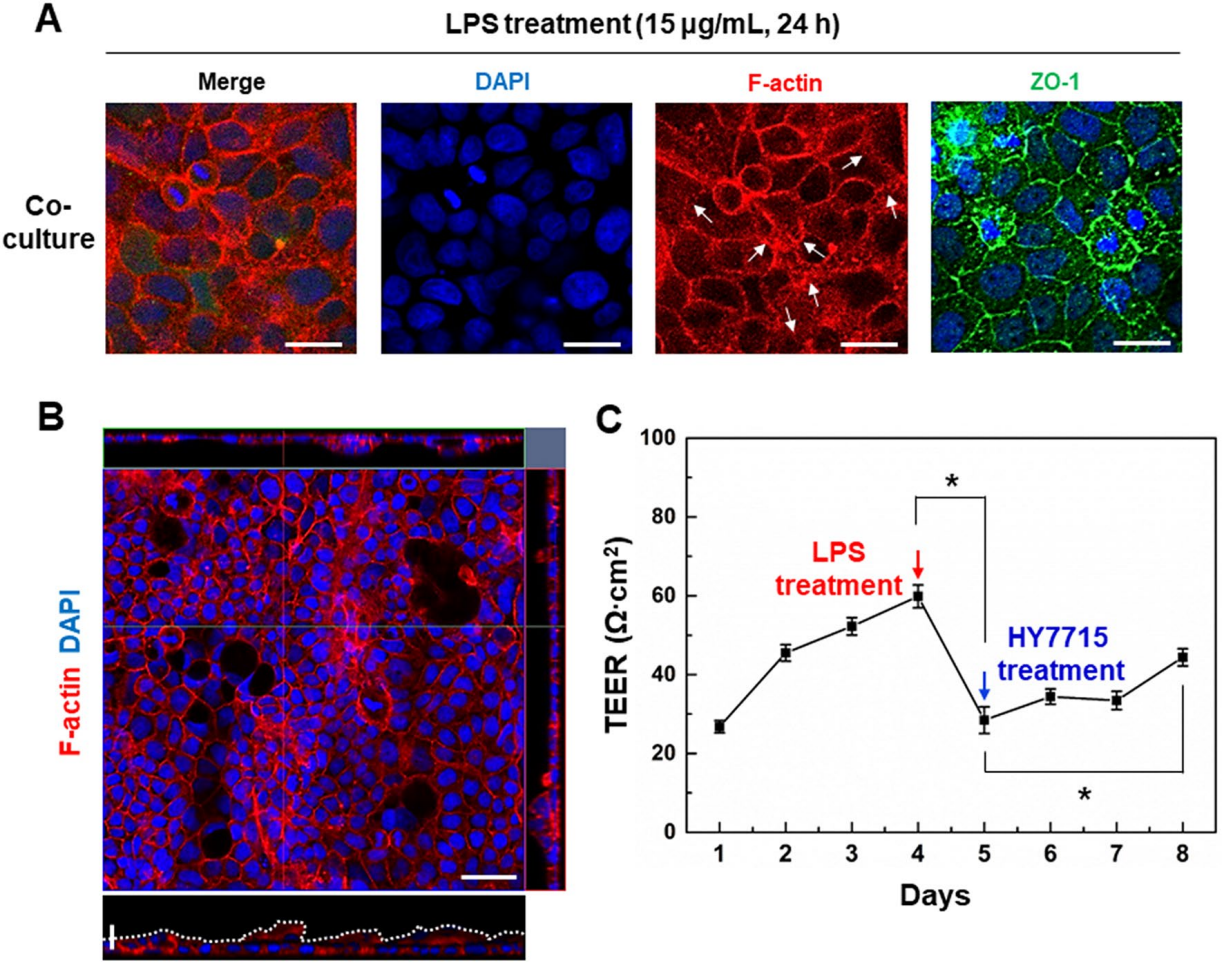
Induction of pathological intestinal injury induced by non-pathogenic LPS endotoxin and anti-inflammatory effect evaluation of probiotics. **A** Morphological analysis of the intestinal barrier damage in response to LPS addition. Scale bars are 25 μm. **B** Fluorescence confocal micrographs (vertical cross-sectional views) of villi recorded at 5 days after staining with F-actin. Horizontal scale bar is 50 μm and Vertical scale bar is 25 μm. **C** Quantification of the intestinal barrier function evaluated by calculated TEER analysis (*p < 0.05)

We further explored whether our gut-on-a-chip system could be used to mimic the human intestinal inflammation in vitro. Following the establishment of the physiologically relevant gut-on-a-chip, we evaluated whether our model could recapitulate the main characteristics of intestinal inflammation. We focused on LPS as alternative stimulus and chose to directly expose the 5 days cultured gut-on-a-chip model to induce an inflammation-like response for 24 h. LPS, is a heat-stable toxin associated with the outer membranes of gram-negative bacteria, belongs to the most studied pathogen-associated molecular patterns and generally presents in the intestinal lumen known for its involvement in intestinal inflammation [55]. In a co-cultured model on a gut-on-a-chip exposed to LPS, we observed more disorganized structure and fainter staining of F-actin structures as compared to non-LPS treated models (Fig. 7A, F-actin, white arrows). The location and distribution of the ZO-1 protein was determined by immunofluorescence assay. Under normal conditions, ZO-1 proteins were localized at the cell membrane and appeared as a continuous band encircling the cells at the cellular borders (Fig. 4.) LPS (15 μg/mL) disturbed the distribution of ZO-1 proteins at the cellular borders. LPS also induced obvious cytoplasmic accumulation of ZO-1 in Caco-2 cells (Fig. 7A, ZO-1 staining). We measured villus heights after LPS administration, because the villus contraction was typically utilized as a measure of small intestinal damage. In co-cultured intestinal models on a gut-on-a-chip at 24 h after LPS administration, the mean villus height was reduced by 32.9%, indicating 24.84 ± 0.73 μm as compared to villi from non-treated LPS co-cultured intestinal models (37.04 ± 2.38 μm, ***p < 0.001) (Fig. 7B). Furthermore, the paracellular permeability was measured by TEER analysis. Administration of 15 μg/mL LPS resulted in a significant decrease in TEER (non-treated LPS and LPS was 59.83 ± 1.68 and 28.44 ± 1.96 Ω∙cm^2^, respectively, *p < 0.05, Fig. 7C). We further evaluated the protective effect of probiotics HY7715 probiotic on LPS-induced epithelial barrier dysfunction. As seen in Fig. 7C, when differentiated Caco-2 cell barriers were treated with 1 × 10^8^ CFU/mL, the TEER analysis was significantly increased as compared to HY7715 probiotic-treated group after LPS treatment at day 8 (LPS and HY7715 probiotic was 28.44 ± 1.96 and 44.39 ± 1.25 Ω∙cm^2^, respectively, *p < 0.05). These results suggested that the epithelium layer differentiated from Caco-2 cells was damaged by LPS treatment in the co-cultured model on a gut-on-a-chip. Its similar trends were observed in other in vitro gut inflammation models [56] and inflammatory bowel disease (IBD) patients [57]. Additionally, the strain HY7715 probiotic suppressed LPS-induced decreases in TEER analysis on a co-cultured intestinal models. It suggests that strain HY7715 probiotic can play an important role in changes in intestinal cell permeability. This result is consistent with the finding that probiotic strains of bacteria, including *Lactobacillus rhamnosus GG* (LGG), have been reported to elevate intestinal epithelial integrity [58] in vitro and improve intestinal barrier function in a human [59]. The previous studies have reported that *L. plantarum* significantly reduced the production of inflammatory cytokines and gut permeability in an IBD pathology by regulating the LPS [60]. This result demonstrated that intestinal epithelial integrity significantly increased in the presence of HY7715 probiotic co-cultures. The presence of the probiotics clearly provides useful microenvironmental signals that enhance epithelial cell functions, which are necessary to maintain this dynamic interface.

## Conclusions

The human microbiomeplays a crucial role in treating the intestinal health and disease. In our gut-on-a-chip system with microelectrode arrays, we cultured the human epithelial cells in a left channel and endothelial cells in a right channel, allowing to simulate and study the impact of fluidic flow and endothelial cells on epithelial cell differentiation without the influence of physical cell contact. Additionally, our gut-on-a-chip co-culture system enables to the perfusion-based cell culture with microbiomes and analyzes the effect of microbiomes to intestinal epithelial barrier functions in vitro. Therefore, our human intestinal gut-on-a-chip system to investigate host-microbe interaction could be a potentially powerful tool for pharmaceutical applications.

## Data Availability

The authors have no data to share since all data are shown in the submitted manuscript.

## Abbreviations

IBD: Inflammatory bowel disease
Caco-2: Human intestinal epithelial cells
LPS: Lipopolysaccharide LPS
PDMS: Polydimethylsiloxane
HUVECs: Human umbilical vein endothelial cells
TEER: Transepithelial electrical resistance
MUC2: Mucin 2

## References

[1] Li XG, Sui WG, Yan HC, Jiang QY, Wang XQ (2014). Animal.

[2] Atarashi K (2017). Science.

[3] Agace WW, McCoy KD (2017). Immunity.

[4] Soderholm AT, Pedicord VA (2019). Immunology.

[5] Puzan M, Hosic S, Ghio C, Koppes A (2018). Sci Rep.

[6] Kinross JM, Darzi AW, Nicholson JK (2011). Genome Med.

[7] Morgan XC (2012). Genome Biol.

[8] Zhao L (2013). Nat Rev Microbiol.

[9] Erny D (2015). Nat Neurosci.

[10] Bartfeld S (2016). Dev Biol.

[11] Patterson DM, Shohet JM, Kim ES (2011). Curr Protoc Pharmacol.

[12] Rowland M (1972). J Pharm Sci.

[13] Hugenholtz F, de Vos WM (2018). Cell Mol Life Sci.

[14] Hubatsch I, Ragnarsson EGE, Artursson P (2007). Nat Protoc.

[15] Dosh RH, Jordan-Mahy N, Sammon C, Le Maitre CL (2018). Tissue Eng Part B Rev.

[16] Park GS, Park MH, Shin W, Zhao C, Sheikh S, Oh SJ, Kim HJ (2017). Stem Cell Rev Rep.

[17] Kasper JY, Hermanns MI, Cavelius C, Kraegeloh A, Jung T, Danzebrink R, Unger RE, Kirkpatrick CJ (2016). Int J Nanomed.

[18] Wang YL, Gunasekara DB, Reed MI, DiSalvo M, Bultman SJ, Sims CE, Magness ST, Allbritton NL (2017). Biomaterials.

[19] Sato T (2009). Nature.

[20] Sato T (2011). Gastroenterology.

[21] Lim J, Ching H, Yoon JK, Jeon NL, Kim Y (2021). Nano Converg.

[22] Lee HN, Choi YY, Kim JW, Lee YS, Choi JW, Kang T, Kim YK, Chung BG (2021). Nano Converg.

[23] Duinen V, Trietsch SJ, Joore J, Vulto P, Hankemeier T (2015). Curr Opin Biotechnol.

[24] Bein A, Shin W, Jalili-Firoozinzhad S, Park MH, Sontheimer-Phelps A, Tovaglieri A, Chalkiadaki A (2018). Cell Mol Gastroenterol Hepatol.

[25] Shah P (2016). Nat Commun.

[26] Bhatia SN, Ingber DE (2014). Nat Biotechnol.

[27] Shin W, Kim HJ (2018). Proc Natl Acad Sci USA.

[28] Huh D, Matthews BD, Mammoto A, Montoya-Zavala M, Hsin HY, Ingber DE (2010). Science.

[29] Maynard CL, Elson CO, Hatton RD, Weaver CT (2012). Nature.

[30] Kim HJ, Huh D, Hamilton G, Ingber DE (2012). Lab Chip.

[31] Kim HJ, Ingber DE (2013). Integr Biol.

[32] Park JY, Hwang CM, Lee SH, Lee SH (2007). Lab Chip.

[33] Haorah J, Schall K, Ramirez SH, Persidsky Y (2008). Glia.

[34] Watson PM, Paterson JC, Thom G, Ginman U, Lundquist S, Webster CI (2013). BMC Neurosci.

[35] van der Helm MW, Odijk M, Frimat JP, van der Meer AD, Eijkel JCT, van den Berg A, Segerink LI (2016). Biosens Bioelectron.

[36] Ainslie KM, Garanich JS, Dull RO, Tarbell JM (2005). J Appl Physiol (1985).

[37] Haegland H, Dahle HK, Eigestad GT, Lie KA, Aavatsmark I (2007). Adv Water Resour.

[38] Toner PG, Carr KE (1969). J Pathol.

[39] Navabi N, McGuckin MA, Linden SK (2013). PLoS ONE.

[40] Johansson ME, Sjovall H, Hansson GC (2013). Nat Rev Gastroenterol Hepatol.

[41] Ding QM, Ko TC, Evers BM (1998). Am J Physiol.

[42] Pageot LP, Perreault N, Basora N, Francoeur C, Magny P, Beaulieu JF (2000). Microsc Res Tech.

[43] Liu J, Williams B, Frank D, Dillon SM, Wilson CC, Landay AL (2017). J Immunol.

[44] Meunier V, Bourrie M, Berger Y, Fabre G (1995). Cell Biol Toxicol.

[45] Marziano M (2019). Biochim Biophys Acta Gen Subj.

[46] Benson K, Cramer S, Galla HJ (2013). Fluids Barriers CNS.

[47] Siddharthan V, Kim YV, Liu S, Kim KS (2007). Brain Res.

[48] Galbraith CG, Skalak R, Chien S (1998). Cell Motil Cytoskeleton.

[49] Bossink E, Zakharova M, de Bruijn DS, Odijk M, Segerink LI (2021). Lab Chip.

[50] Beduneau A, Tempesta C, Fimbel S, Pellequer Y, Jannin V, Demarne F, Lamprecht A (2014). Eur J Pharm Biopharm.

[51] Servin AL, Coconnier MH (2003). Best Pract Res Clin Gastroenterol.

[52] Vantrappen G, Janssens J, Hellemans J, Ghoos Y (1977). J Clin Invest.

[53] Schillinger U, Guigas C, Holzapfel WH (2005). Int Dairy J.

[54] Gopal PK, Prasad J, Smart J, Gill HS (2001). Int J Food Microbiol.

[55] Guo S, Al-Sadi R, Said HM, Ma TY (2013). Am J Pathol.

[56] Park JJ, Moon HJ, Park JH, Kwon TH, Park YK, Kim JH (2016). J Neurosurg Spine.

[57] Suenaert P, Bulteel V, Lemmens L, Noman M, Geypens B, Van Assche G, Geboes K, Ceuppens JL, Rutgeerts P (2002). Am J Gastroenterol.

[58] Fang HW, Fang SB, Chiang Chiau JS, Yeung CY, Chan WT, Jiang CB, Cheng ML, Lee HC (2010). J Med Microbiol.

[59] Dai C, Zhao DH, Jiang M (2012). Int J Mol Med.

[60] Chelakkot C, Ghim J, Ryu SH (2018). Exp Mol Med.

